# Investigating the impact of migraine on short-term and working memory: a systematic review and meta-analysis

**DOI:** 10.1007/s00415-026-13672-w

**Published:** 2026-03-17

**Authors:** Kenya McKay, Kate Kelly

**Affiliations:** 1 Department of Psychology, Counselling and Therapy, La Trobe University, Melbourne, VIC Australia; 2 School of Psychology and Public Health, La Trobe University, Melbourne, VIC Australia

**Keywords:** Migraine, Short-term memory, Working memory, Meta-analysis, Systematic review, Cognitive function

## Abstract

**Objective:**

To conduct a systematic review and meta-analysis on past research to ascertain the impact that experiencing migraines has on memory. Specifically, this research explores the short-term and working memory domains and where possible delineates between verbal and visual memory.

**Background:**

Migraine is a prevalent neurological disorder that affects up to 15% of the global population. Migraine is comprised of physical symptoms including head pain, nausea, vomiting, and sensitivity to light and sound. There are also various subtypes that are based on specific symptom manifestations such as the presence of visual aura. Furthermore, a commonly reported cognitive symptom is ‘brain fog’, a colloquial term used to describe everyday cognitive challenges particularly in relation to attention and memory. The empirical literature examining memory performance in migraine has produced inconsistent findings: some studies report clear cognitive impairment, whereas others suggest minimal or no deficits. In addition, research to date has been limited in its examination of specific memory domains and has not consistently considered the influence of migraine characteristics. Thus, this systematic review and meta-analysis aimed to investigate the short-term and working memory performance of migraineurs compared to healthy controls. Subsidiary aims included investigating the memory performance of migraineurs with aura and without aura and investigating the impact of migraine on visual and verbal memory performance.

**Methods:**

A systematic search was conducted in Embase, CINAHL, MEDLINE, PsycINFO, and Web of Science Core Collection. Papers were screened in two stages (titles and abstracts and full texts) using inclusion criteria relevant to the aims. Of 3880 articles extracted, 16 met the criteria for inclusion. Random effects models were conducted in JASP and utilised Hedge’s g as the effect size. Heterogeneity was evaluated using the *Q* and *I*^2^ statistics. Publication bias was examined using Egger’s regression test and funnel plots.

**Results:**

No significant differences were found between migraineurs and healthy controls in short-term memory performance. Specifically, no significant differences were observed between migraineurs with aura and healthy controls, migraineurs without aura and healthy controls, or migraineurs with and without aura. Similarly, no significant differences were observed in short-term visual or verbal memory performance. In contrast, a significant difference was observed in working memory performance, with migraineurs demonstrating poorer performance relative to healthy controls.

**Conclusions:**

The findings of this systematic review and meta-analysis indicate that short-term memory performance is preserved in adults with migraine, whereas working memory shows a selective impairment. This pattern suggests that migraine is not associated with a global memory deficit, but rather with vulnerability in higher-order memory processes requiring active manipulation and cognitive control. These results help reconcile objective cognitive findings with commonly reported experiences of brain fog and suggest that migraine-related cognitive complaints may reflect difficulties in cognitive efficiency rather than fundamental memory storage deficits during the interictal period.

**Trial registration policy:**

There was no registration as this paper is not experimental.

**Supplementary Information:**

The online version contains supplementary material available at 10.1007/s00415-026-13672-w.

## Introduction

Migraine is one of the most common and disabling neurological disorders worldwide, affecting approximately 14–15% of the global population and ranking as a leading cause of neurological disability [1, 2]. Migraine disproportionately affects women, with epidemiological studies consistently reporting a female-to-male ratio of approximately 3:1, particularly during reproductive years [2]. Beyond pain, migraine involves a range of sensory, cognitive, and functional symptoms that can disrupt daily life [1]. One frequently reported cognitive symptom is “brain fog”, a colloquial term for difficulties such as mental fatigue, problems concentrating, and forgetfulness [3–5]. Although not part of diagnostic criteria, brain fog is increasingly recognised as a meaningful aspect of the migraine experience and can occur across multiple phases of the migraine cycle [4, 6, 7]. Whether these subjective experiences reflect measurable memory disturbances remains unclear, contributing to ongoing uncertainty in clinical practice.

Migraine attacks typically last 4–72 h and involve moderate to severe, often unilateral pulsating pain accompanied by nausea, vomiting, photophobia, and phonophobia [8]. Attacks are usually classified as migraine without aura (MwoA) or with aura (MwA), where aura refers to transient neurological disturbances, most commonly visual phenomena, occurring before or during the headache phase [8]. These attacks progress through premonitory, aura, headache, and postdrome phases, often grouped into interictal (premonitory, postdrome) and ictal (aura, headache) periods [9–11]. While research commonly assesses patients during the interictal phase, reports of brain fog throughout the cycle raise questions about whether migraine contributes to measurable cognitive disruption.

Memory is a core cognitive function supporting learning, reasoning, and everyday activities [12]. Short-term memory (STM) involves temporary storage of information and is a foundational cognitive skill, whereas working memory (WM) is more complex and involves the active manipulation of that information [13]. These processes draw on limited cognitive resources that may be disrupted by pain, fatigue, and sensory overload, factors commonly present in migraine [13–15]. Memory can also be considered by modality, with distinct subsystems supporting visual and verbal information [12, 16]. Although brain fog is a broad and heterogeneous construct encompassing mental fatigue, attentional fluctuation, and reduced cognitive efficiency, short-term and working memory represent theoretically central targets for investigation [5]. Together, STM and WM support the temporary maintenance and manipulation of information underpinning higher-order cognition, including reasoning, language comprehension, and goal-directed behaviour [12, 14]. Disruption at this level would be expected to cascade into broader subjective cognitive difficulties [12]. Accordingly, many empirical studies investigating migraine-related cognitive complaints have operationalised brain fog through STM and WM paradigms, particularly during interictal periods when standardised neuropsychological assessment is feasible [17–22]. Given the high prevalence of brain fog reports and established links between pain and cognitive load, clarifying whether objective STM and WM deficits occur in migraine is clinically important.

Existing research offers conflicting conclusions. Some studies report global cognitive deficits, including in STM, WM, attention, and executive function [17–19], and meta-analyses of broader cognition also suggest impairment, although memory is often treated as a single domain [18, 20]. Other studies report memory deficits across domains and modalities [17, 21–23], sometimes in both MwA and MwoA groups [23–25], although comorbidities are not always adequately controlled [18, 26]. In contrast, several studies report comparable STM and WM performance between migraine and control groups [20, 27, 28], including in older adults, suggesting possible adaptive mechanisms that preserve cognition [29, 30]. A smaller body of work has even found superior memory performance in migraine, such as enhanced visual STM in MwoA [27] and better recall of emotional words [59]. Methodological differences in migraine subtype, symptom profiles, and cognitive measures likely contribute to these discrepancies.

Together, this literature highlights the need for a focussed synthesis that considers migraine subtype, memory domain, and modality. Clarifying whether objective memory impairment exists, and under what conditions, is important for understanding the mechanisms underlying brain fog, supporting patient care, and guiding future research.

### The present study

With prevalence rates rising and diagnostic criteria becoming more refined, understanding the nature of the migraine experience is key in developing management strategies. Brain fog is a major symptom for migraineurs, and whilst recognised as a subjective experience, may suggest a connection to memory impairment [5]. Despite numerous studies exploring the impact of migraine on memory, there is no explicit conclusion, solidifying the rationale for a meta-analytic approach to the topic.

By approaching STM and WM performance in migraineurs in a systematic manner, the present study aims to synthesise the existing literature to gain a more comprehensive understanding of the impact of migraine on memory. Specifically, this systematic review and meta-analysis aims to investigate the objective STM and WM performance of migraineurs compared to healthy controls. As STM is a foundational cognitive skill underpinning many higher order functions and working memory is a more complex executive process, investigating these systems may elicit insight into the objective cognitive skills that may explain the experience of brain fog in migraineurs. Subsidiary aims include investigating the objective memory performance of individuals who experience MwA and those who experience MwoA, and finally, this study aims to investigate the impact of migraine on the visual and verbal memory performance of migraineurs compared to healthy controls.

## Methodology

This systematic review and meta-analysis was conducted in accordance with PRISMA guidelines [31]. For transparency, the PRISMA checklist is displayed in supplementary Table 1.

This project was registered with Open Science Framework (10.17605/OSF.IO/4BZKV).

### Search strategy

A systematic search of Embase, CINAHL, MEDLINE, PsycINFO, and Web of Science Core Collection was conducted on 26 May 2025 and 6 months later on 20 November 2025. Embase, MEDLINE, and Web of Science Core Collection were selected as core databases due to their broad coverage [32], with CINAHL and PsycINFO added to capture allied health and clinical psychology literature. Search terms combined migraine-related keywords with terms for STM, WM, and visual and verbal memory (see Table 1). The search strategy was developed using prior literature and refined in consultation with two academic librarians. Once finalised, all databases were searched using the same key terms and query strings outlined in Table 1.

**Table 1:** Overview of search strategy

Search strategy	
1	“migraine” OR “chronic headache” OR “daily headache” OR “persistent headache”
2	“memory” OR “short term memory” OR “working memory” OR “STM” OR “WM” OR “visual memory” OR “verbal memory” OR “visuospatial” OR “phonological loop” OR “visuospatial sketchpad”
3	1 and 2

### Study selection

A hierarchy of eligibility criteria was established to ensure the inclusion of studies relevant to the research aims (see Table 2). All studies included were required to meet all criteria.

**Table 2:** Hierarchy of inclusion criteria

Inclusion criteria	
1	Available in english
2	Is an original, experimental study (not a conference paper, dissertation, review)
3	Available as a full text
4	Participants are adults (aged between 18 and 55)
5	Study contains a migraine or headache group/s and a healthy control group: participants in the migraine or headache group have experienced or are experiencing intense, throbbing pain in the head, nausea and sensitivity to light and sound (or a mix of symptoms) OR are diagnosed according to ICHD-3
6	Participants do not have comorbid conditions that would impact cognitive performance
7	The method utilises at least one standardised objective assessment of STM or WM
8	Outcome data includes means and standard deviations of the performance on memory assessments

Search results were imported into Covidence, which was used to identify and remove duplicates and manage screening. Title and abstract screening were followed by full-text review, with both stages conducted independently by the student researcher and supervising researcher. Discrepancies were resolved through discussion.

### Data extraction and synthesis

A Microsoft Excel template was developed to facilitate data extraction. Data extracted included paper characteristics (author/s, title, year, aim/s), participants details (sample size, age, sex split, migraine subtype), and memory assessment information (task name, stimulus characteristics, memory domain, modality, outcome data). Any conflicts were resolved through discussion or reconfirmation of data. Where memory tasks involved multiple cognitive processes (e.g. learning, visuo-construction, or executive demands), classification followed the dominant use of the task within the neuropsychological literature. This approach was adopted to ensure consistency across studies whilst acknowledging that some measures may not map exclusively onto a single cognitive domain.

### Quality assessment and risk of bias

Risk of bias and quality assessment were assessed using the standard quality assessment criteria for evaluating primary research papers from a Variety of Fields [33]. Items specific to intervention studies were removed, resulting in an 11-item checklist (maximum score = 22). Each study was independently rated by both researchers, with disagreements resolved using the more conservative score. Summary scores were converted to percentages and classified as limited (0–25%), adequate (26–50%), good (51–75%), or strong (76–100%). If papers scored limited–adequate, they would be excluded from the study.

### Data analysis

All data were analysed in Jeffrey’s Amazing Statistics Program (JASP) using a random effects model that requires a minimum of five studies to meet power (see Table 3).

**Table 3:** Variables and data points of meta-analyses conducted

Variables in meta-analysis	Number of data points
STM performance: Migraineurs compared to healthy controls	26
WM performance: Migraineurs compared to healthy controls	8
STM performance: MwA compared to healthy controls	8
STM performance: MwoA compared to healthy controls	11
STM performance: MwA compared to MwoA	8
STM-visual performance: migraineurs compared to healthy controls	8
STM-verbal performance: migraineurs compared to healthy controls	18

Effect sizes and standard errors were calculated in MAVIS [34] using Hedges’ *g*, which is preferred for smaller samples [35]. Effect sizes were interpreted using Cohen’s guidelines (small = 0.2, medium = 0.5, large = 0.8) [36]. Ninety-five per cent confidence intervals were calculated and displayed in forest plots.

Between-study heterogeneity was assessed using *Q* and *I*^2^ statistics, with *p* < 0.10 and higher *I*^2^ values indicating greater heterogeneity. Egger’s regression test was used to evaluate potential publication bias and, where more than 10 studies were present, this was supplemented with funnel plots. For any significant pooled effect, Rosenthal’s Fail-Safe *N* was used to estimate the number of additional null studies required to reduce the effect to non-significance.

## Results

### Study selection

**Fig. 1:**
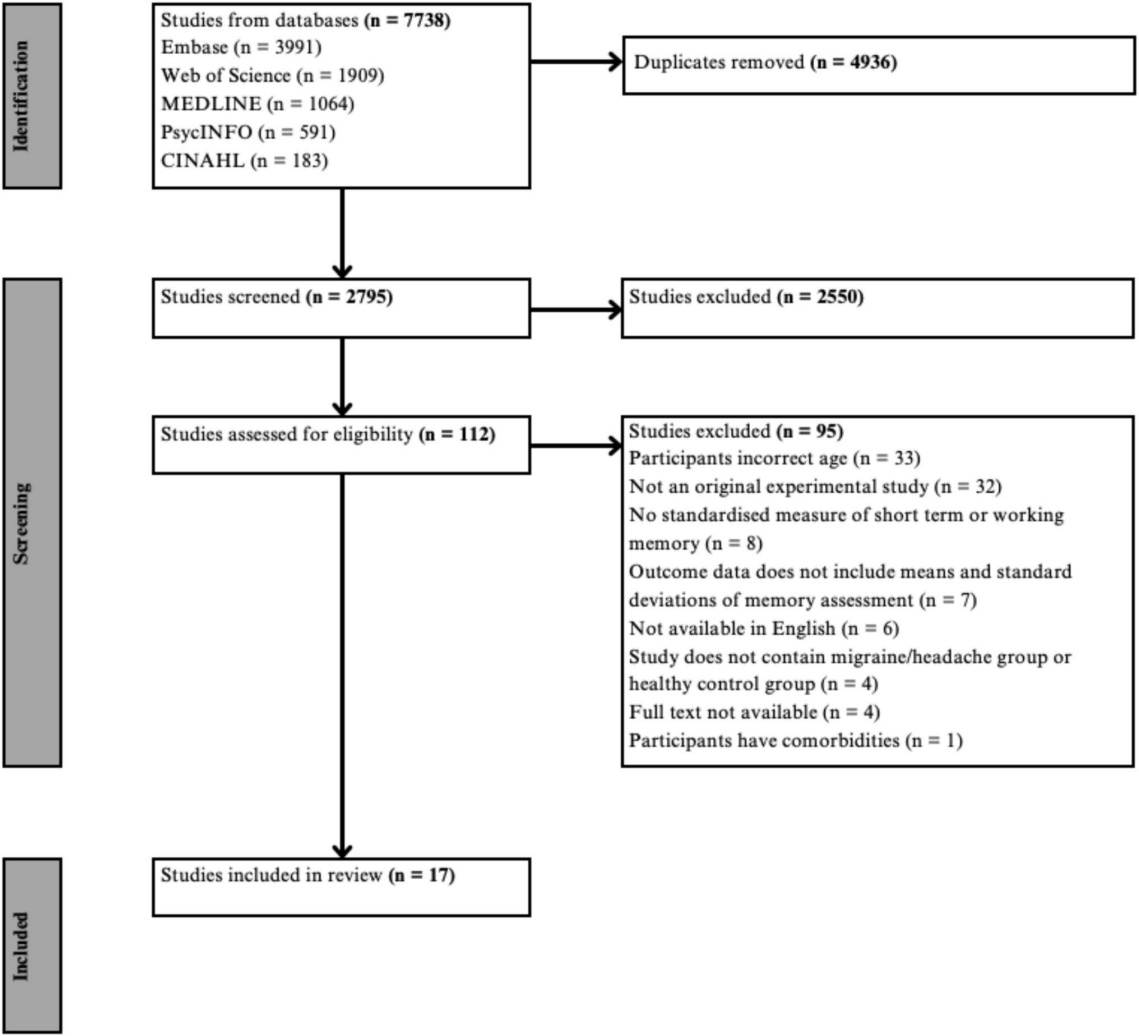
PRISMA flowchart

A total of 7738 articles were imported from the databases into Covidence. 4943 duplicates were identified and removed, leaving 2795 articles that underwent title and abstract screening. This phase resulted in conflicts in 6% of responses from the reviewers. 112 articles’ full texts were retrieved and screened. This phase resulted in conflicts in 5% of responses from reviewers. Finally, 95 studies were excluded, leaving 17 eligible for analysis. Of the 95 studies excluded, 6 were not available in English, 32 were not the appropriate study design, 4 did not have the full text available, 33 were outside the age range, 4 did not include a migraine group and healthy control group, 1 included participants with comorbidities, 8 did not include a standardised measure of STM or WM, and 7 did not include the appropriate outcome data (means and standard deviations). Figure 1 depicts the extraction and screening process.

### Study characteristics

This review had a total of 1483 participants across all extracted studies. 781 were migraineurs and 702 were healthy controls. Sample sizes ranged from 19 to 100 for the migraine groups and 14 to 150 for the healthy controls. Of the total, 1157 participants had their sex reported, of which 960 (82.97%) were female (migraineurs = 515, healthy controls = 445). Table 4 depicts the characteristics for participants and memory assessments extracted across all 17 studies.

**Table 4:** Participant and memory assessment characteristics

Study	Characteristics of the migraine group			Characteristics of the control group			Characteristics of the memory test		
	Sample size	Sex split M:F	Age—mean (SD)	Sample Size	Sex split M:F	Age—mean (SD)	Name of task/s	Type of memory measured	Modality of information
Baschi et al., 2019	21	9:12	29 (4.32)	21	9:12	27.90 (3.16)	Buschke selective reminding test	STM	Verbal
							Corsi block tapping test	STM	Visual
Burker et al., 1989	47	0:47	19.32 (1.4)	24	0:24	18.66 (1.09)	Rey-Osterrieth complex figure test	STM	Visual
Chowdhury et al., 2024	90	15:65	28.30 (7.8)	150	36:114	28.90 (4.60)	Digit span (Forward)	STM	Verbal
Fathi et al., 2025	41	0:41	31.63 (6.17)	40	0:40	34.18 (5.21)	Benton visual retention test	STM	Visual
							Digit span (Backward)	WM	Verbal
González-Mingot et al., 2022	46		47.17	50		47.82	Rey-Osterrieth complex figure test	STM	Visual
Huang et al., 2017	34	6:28	36.07 (10.05)	24	6:18	36.05 (12.97)	Montreal cognitive assessment	STM	Verbal
							Rey-Osterrieth complex figure test	STM	Visual
Le Pira et al., 2000	30	4:26	33.23 (12.20)	14	2:12	33.86 (12.45)	California verbal learning test	STM	Verbal
							California learning test	STM	Verbal
							Corsi block tapping test	STM	Verbal
							Digit span (Forward)	STM	Verbal
							Digit span (Backward)	WM	Verbal
							Rey-Osterrieth complex figure test	STM	Visual
Lo Buono et al., 2019	100		39.67 (12.96)	50		38.16 (11.32)	Rey Auditory verbal learning test	STM	Verbal
McKendrick et al., 2006	29	11:18		27	9:18		Repeatable battery for the assessment of neuropsychological status	STM	Verbal
Mulder et al., 1999	30	4:26	24.71 (3.61)	30			Pattern memory	STM	Visual
Quiroz-Padilla et al., 2016	40	9:31	26.2 (6.06)	23	8:15	24 (5)	Grober and Buschke free and cued selective reminding test	STM	Verbal
							Spain—complutense verbal learning test	STM	Verbal
							Rey-Osterrieth complex figure test	STM	Visual
Romigi et al., 2008	20		37.70 (13.94)	20		37.50 (12.47)	Rey auditory verbal learning test	STM	Verbal
							Rey-Osterrieth complex figure test	STM	Visual
							Digit span (Forward)	STM	Verbal
							Digit span (Backward)	WM	Verbal
Santangelo et al., 2016	72	9:63	34.90 (11.20)	72	6:66	33.80 (11.90)	Montreal cognitive assessment	STM	Verbal
Tomé-Pires & Miró et al., 2014	35	4:31	30 (8.10)	31	12:19	24 (4)	Memory recall of word	STM	Verbal
Tunç et al., 2018	100	9:91	36.70 (9.40)	80	10:70	34.40 (11.02)	Montreal cognitive assessment	STM	Verbal
Zeitlin & Oddy, 1984	19	3:16	36.30	19	3:16	35.30	Paced auditory serial additional test	WM	Verbal
Zhang et al., 2012	27	7:20	33.60 (10.41)	27	6:21	31.96 (8.78)	Digit span (Forward)	STM	Verbal
							Digit span (Backward)	WM	Verbal

### Quality assessment

An assessment of the 17 papers included in this meta-analysis was undertaken to examine potential risk of bias and quality. The standard quality assessment criteria for evaluating primary research papers from a variety of fields checklist was utilised [33]. All 17 papers received a score of 16 or above (*M* = 19.47, SD = 1.46). Of which, 16 were considered of strong quality and 1 of good quality. None of the 17 studies were considered less than adequate; thus, all were retained for analysis. Table 5 presents the results of the quality assessment.

**Table 5:**
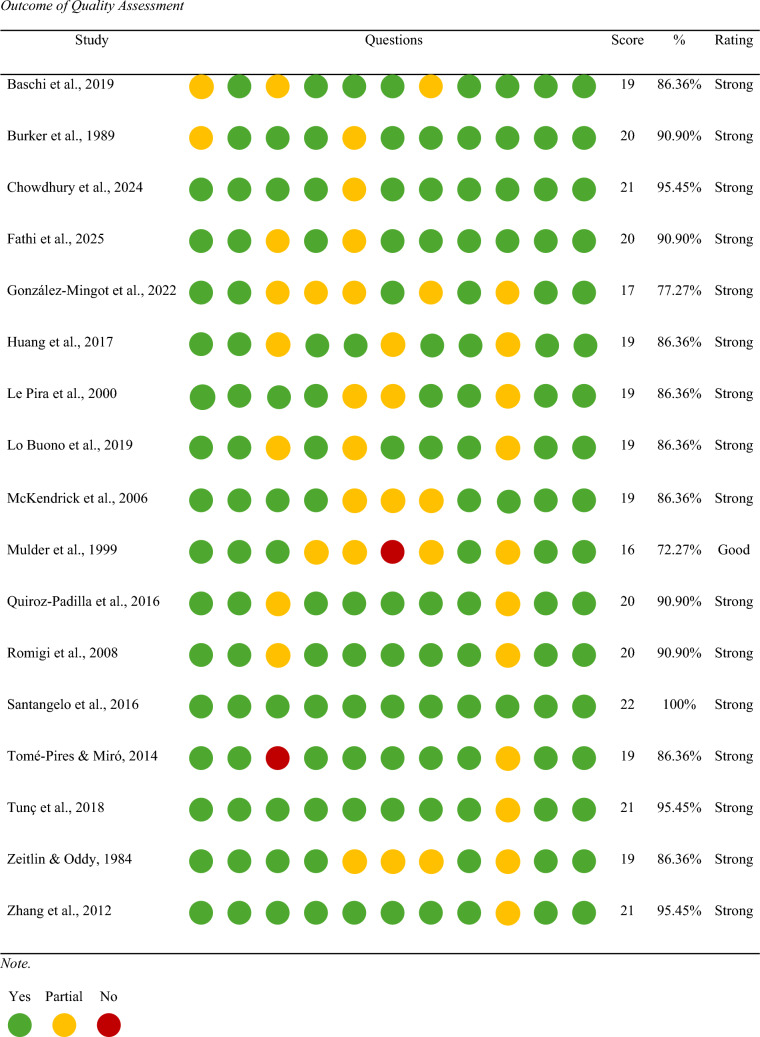
Outcome of quality assessment

### Meta-analysis investigating STM performance in migraineurs compared to healthy controls

**Fig. 2:**
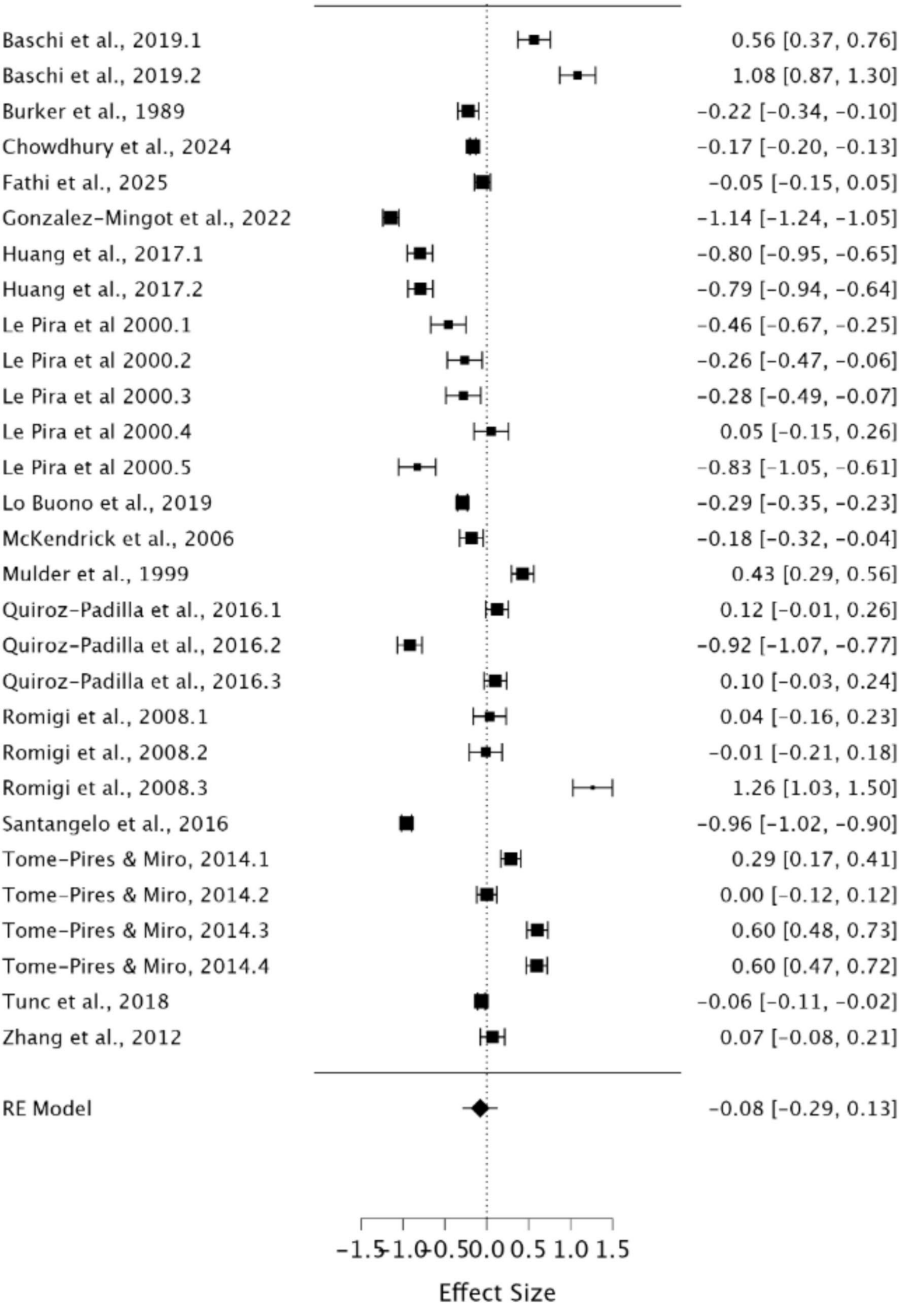
Forest plot of effect sizes and confidence intervals for STM performance in migraineurs compared to healthy controls. *RE* Random effects, *Effect Size* Hedge’s *g*

**Fig. 3:**
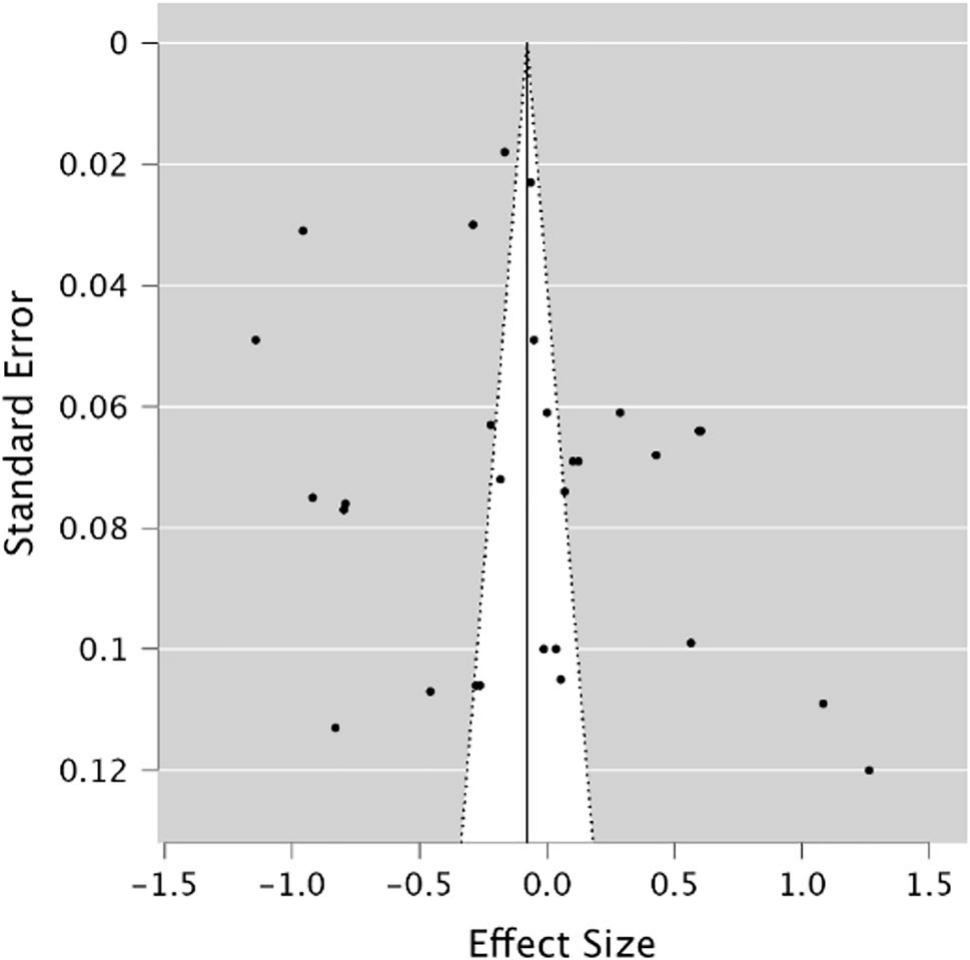
Funnel plot of potential publication bias for STM performance in migraineurs compared to healthy controls

A random effects model was utilised to assess STM in migraineurs compared to healthy controls. As observed in Fig. 2, no significant difference was present between groups, demonstrating a small effect size (*g* = − 0.08, 95% CI − 0.29–0.13, *p* = 0.47). The *Q* statistic was 2162.59 (*df* = 28) and was significant (*p* < 0.001), indicating significant heterogeneity. Heterogeneity was also considered high when using the *I*^2^ statistic (*I*^2^ = 99.18%). Egger’s regression test and visual inspection of the funnel plot (Fig. 3) did not indicate the presence of publication bias (*p* = 0.15).

### Meta-analysis investigating WM performance in migraineurs compared to healthy controls

**Fig. 4:**
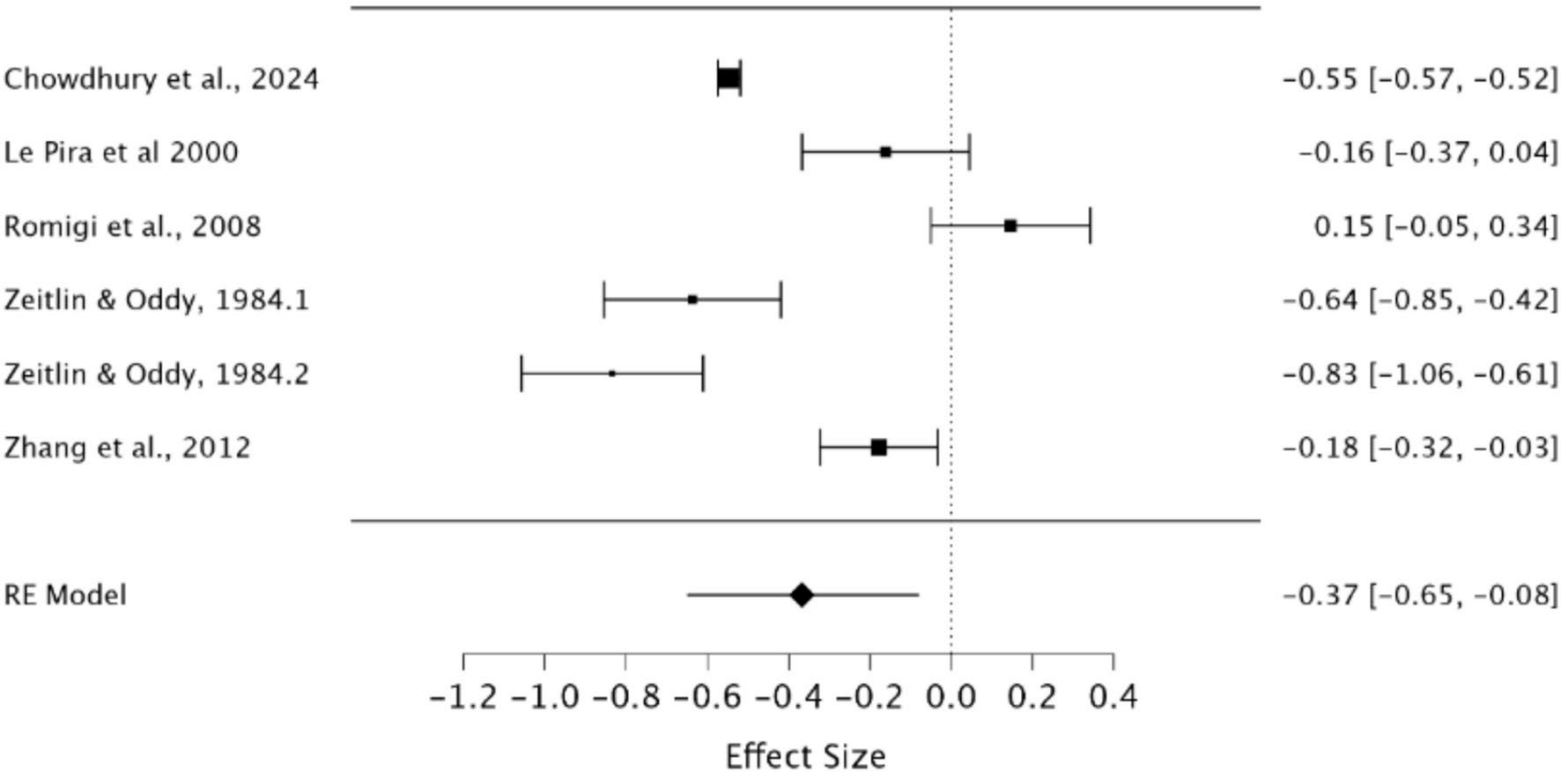
Forest plot of effect sizes and confidence intervals for WM performance in migraineurs compared to healthy controls. *RE* Random effects, *Effect Size* Hedge’s *g*

A random effects model was used to assess WM in migraineurs compared to healthy controls. As observed in Fig. 4, a significant difference was present between groups demonstrating a small effect size (*g* = − 0.37, 95% CI −0.65 – −0.08, *p* = 0.01). Heterogeneity was considered high when using the *I*^2^ statistic (*I*^2^ = 96.09%). Egger’s regression test did not indicate the presence of publication bias (*p* = 0.93). Rosenthal’s Fail-Safe *N* indicated that 1094 additional null studies would be needed to render these significant findings non-significant.

### Meta-analysis investigating STM: a migraine subtype-based approach

In line with the study’s aims and given the significant heterogeneity of the initial analyses, subgroup analyses were conducted to determine whether migraine subtype or memory modality contributed to the high heterogeneity observed in the primary analyses. Subgroup analyses investigating migraine subtype were limited to MwA and MwoA due to sample size. These subgroup analyses considered MwA and MwoA separately and comparatively.

**Fig. 5:**
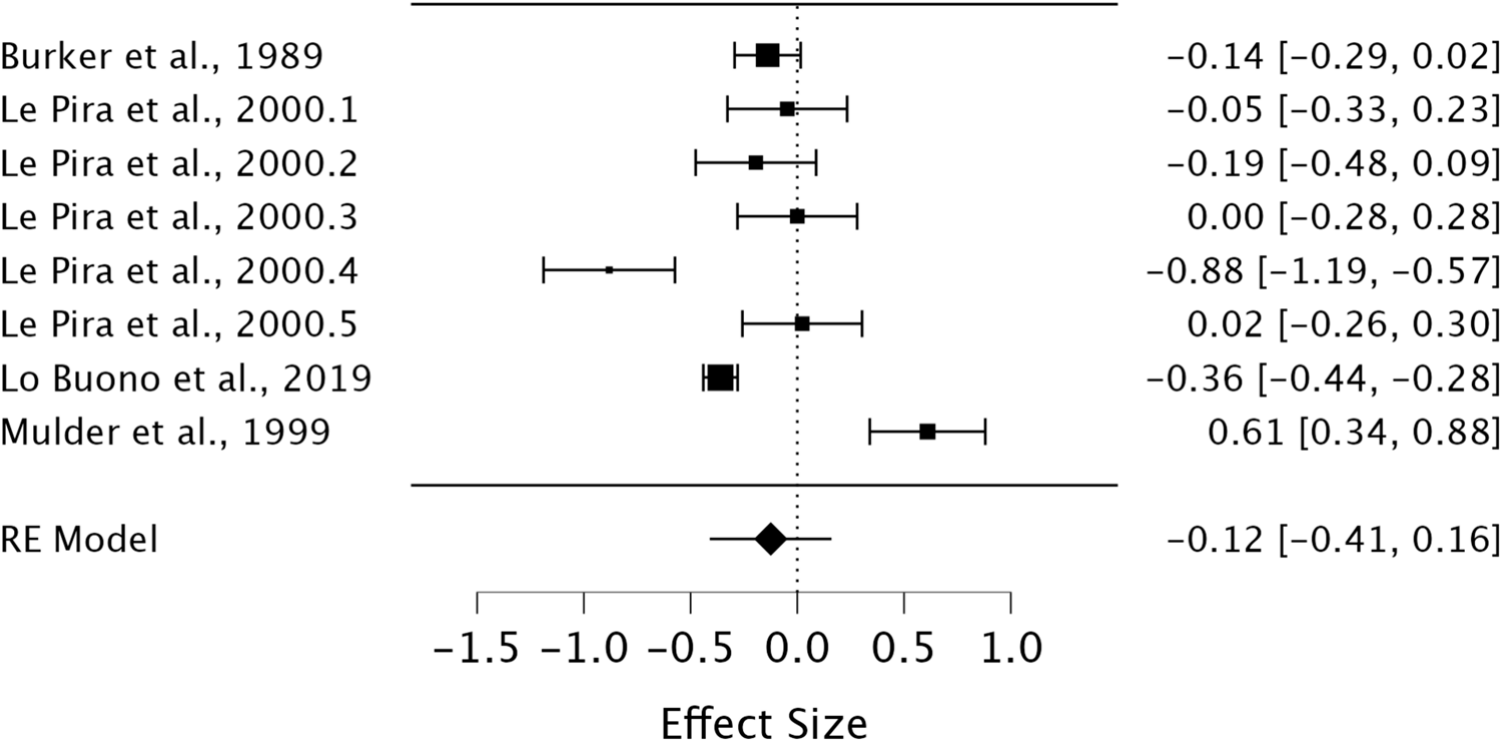
Forest plot of effect sizes and confidence intervals for STM performance in MwA individuals compared to healthy controls. *RE* Random effects, *Effect Size* Hedge’s *g*

A random effects model was utilised to assess STM in MwA individuals compared to healthy controls. As observed in Fig. 5, no significant difference was present between groups demonstrating a small effect size (*g* = − 0.14, 95% CI −0.41–0.16, *p* = 0.39). Heterogeneity was considered high using the *I*^2^ statistic (*I*^2^ = 93.39%). Egger’s regression test did not indicate the presence of publication bias (*p* = 0.79).

**Fig. 6:**
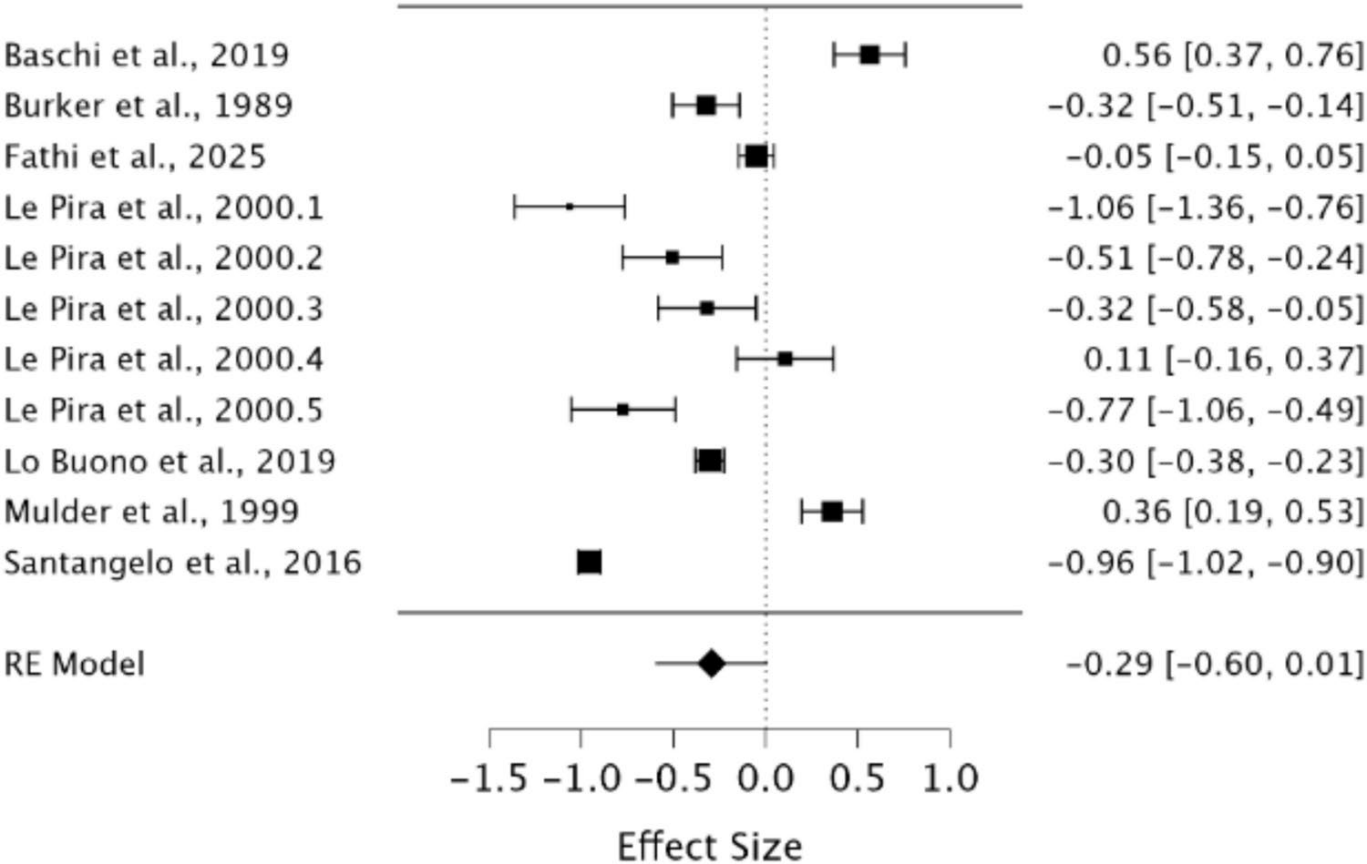
Forest plot of effect sizes and confidence intervals for STM performance in MwoA individuals compared to healthy controls. *RE* Random effects, *Effect Size* Hedge’s *g*

**Fig. 7:**
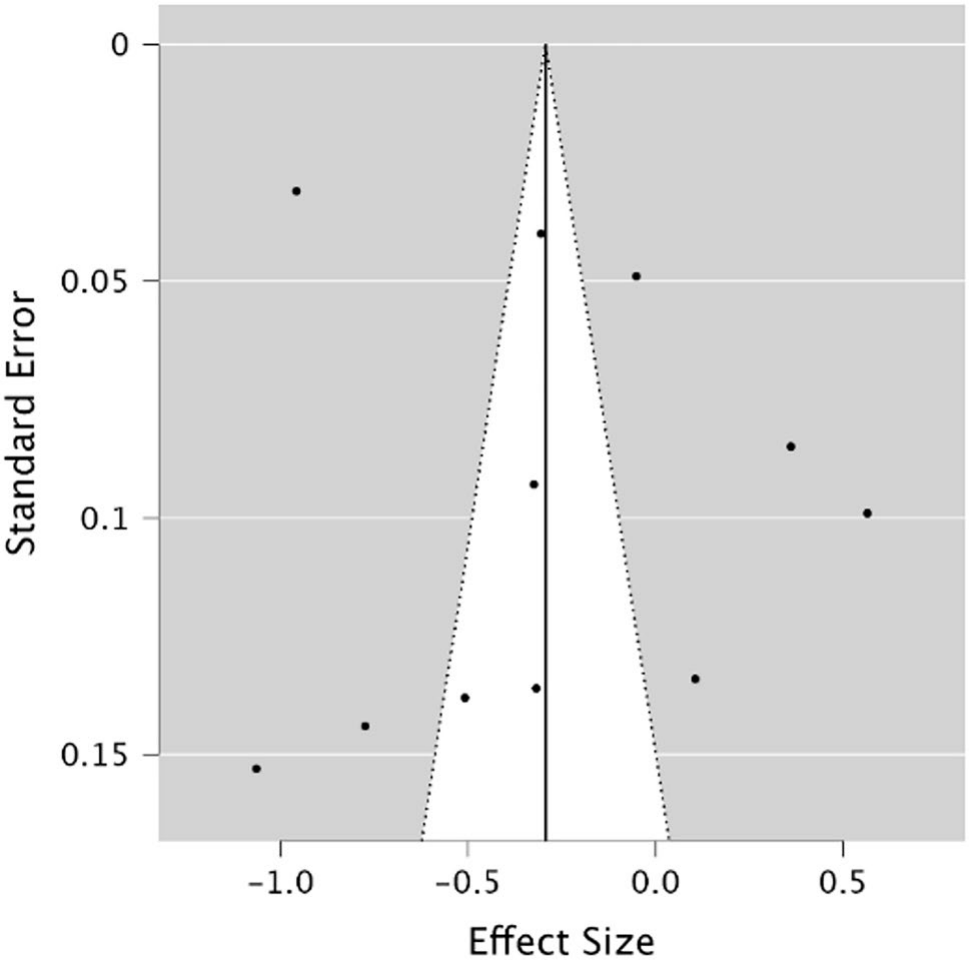
Funnel plot of potential publication bias for STM performance in MwoA individuals compared to healthy controls

A random effects model was utilised to assess STM in MwoA individuals compared to healthy controls. As observed in Fig. 6, no significant difference was present between groups demonstrating a small effect size (*g* = − 0.29, 95% CI −0.60–0.01, *p* = 0.06). The Q statistic was 583.59 (*df* = 10) and was significant (*p* = < 0.001), indicating significant heterogeneity. Heterogeneity was considered high when using the *I*^2^ statistic (*I*^2^ = 98.15%). Egger’s regression test and visual inspection of the funnel plot (Fig. 7) did not indicate the presence of publication bias (*p* = 0.70).

**Fig. 8:**
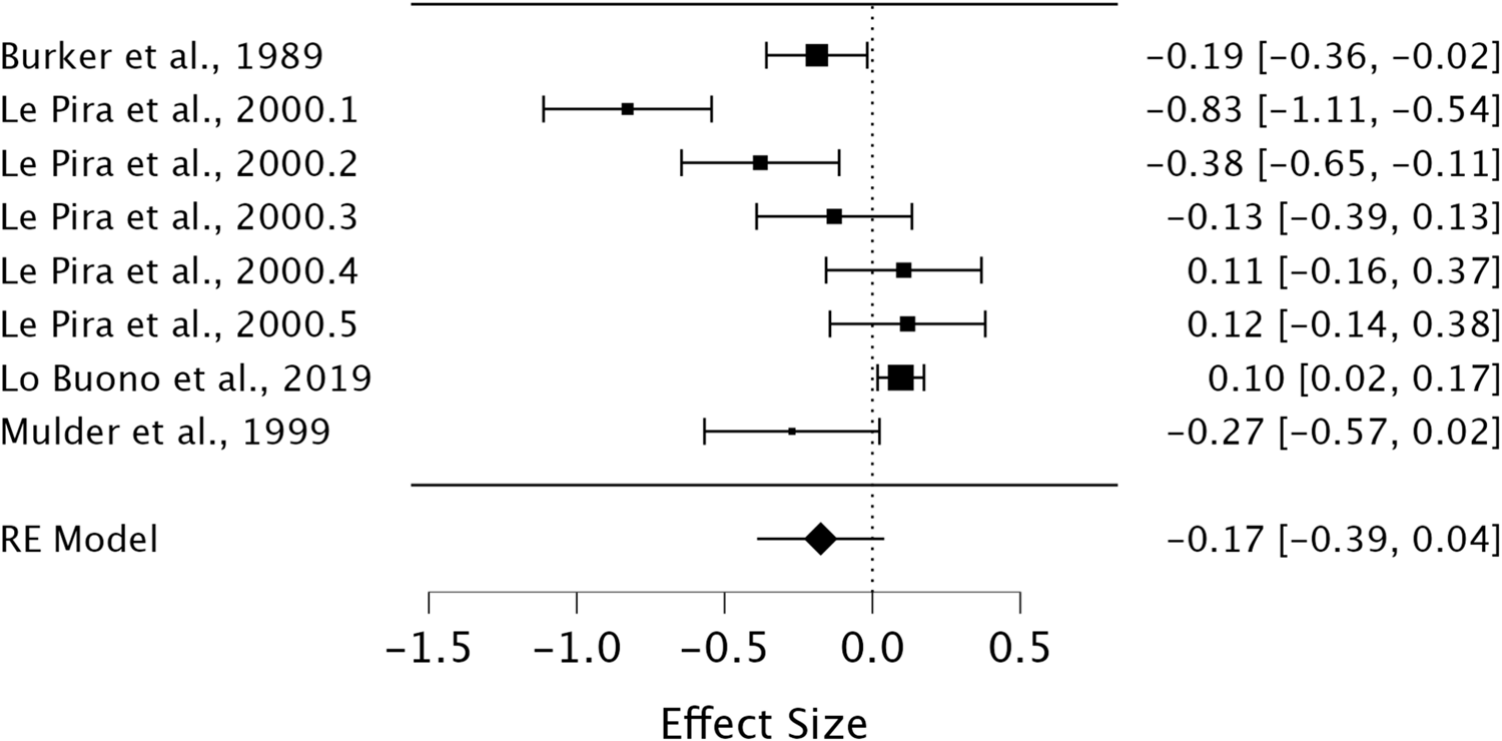
Forest plot of effect sizes and confidence intervals for STM performance in MwA individuals compared to MwoA individuals. *RE* Random effects, *Effect Size* Hedge’s *g*

A random effects model was utilised to assess STM in MwA compared to MwoA individuals. As observed in Fig. 8, no significant difference was present between groups demonstrating a small effect size (*g* = −0.17, 95% CI − 0.39–0.04, *p* = 0.11). Heterogeneity was considered high when using the *I*^2^ statistic (*I*^2^ = 88.81%). Egger’s regression test did not indicate the presence of publication bias (*p* = 0.23).

### Meta-analysis investigating STM: a modality-based approach

The second subgroup analyses aimed to explore visual and verbal memory performance. Subgroup analyses were limited to STM due to sample size.

**Fig. 9:**
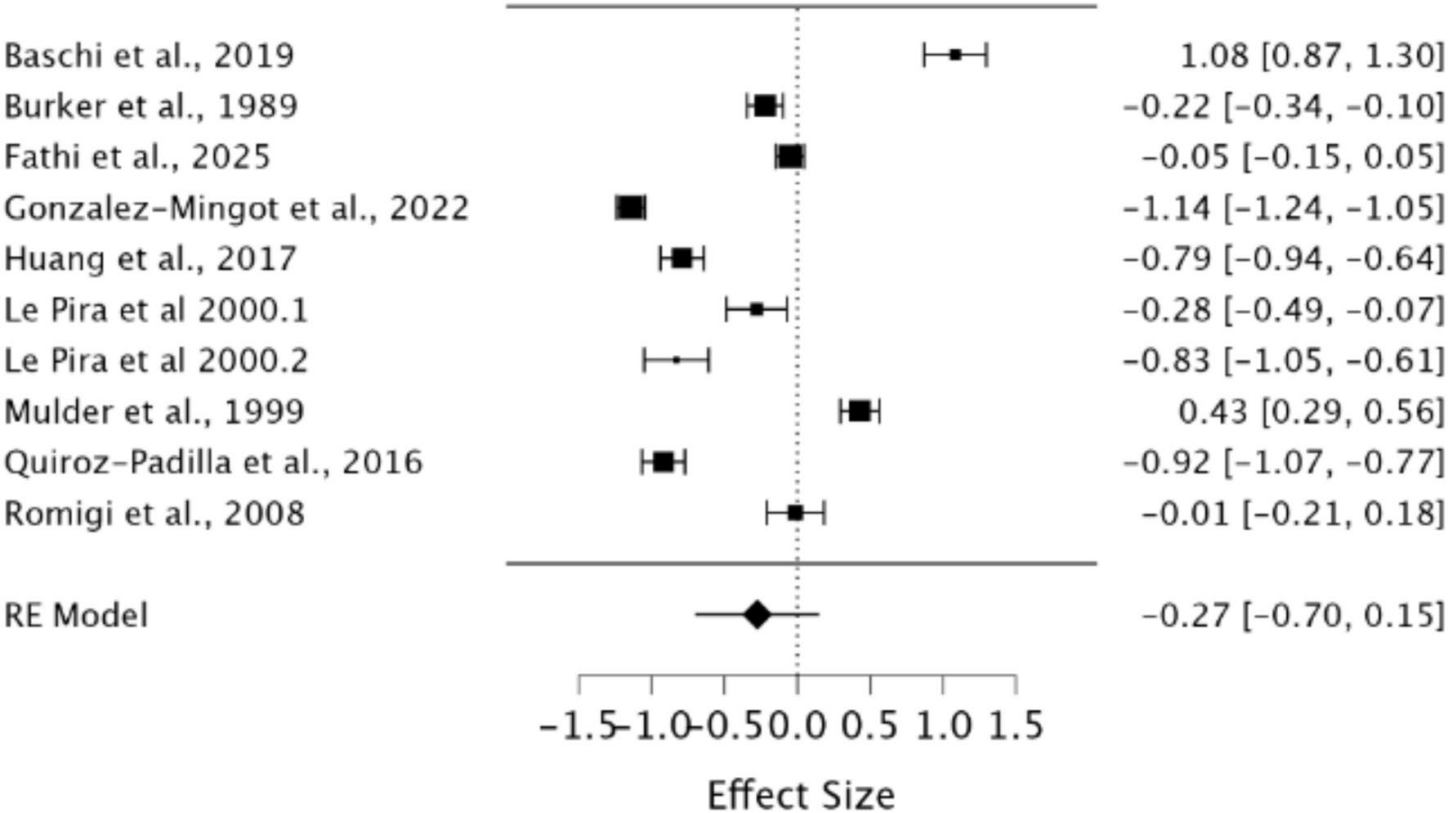
Forest plot of effect sizes and confidence intervals for visual STM performance in migraineurs compared to healthy controls. *RE* Random effects, *Effect Size* Hedge’s *g*

A random effects model was used to assess visual STM in migraineurs compared to healthy controls. As observed in Fig. 9, no significant difference was present between groups demonstrating a small effect size (*g* = − 0.27, 95% CI − 0.70–0.15, *p* = 0.20). Heterogeneity was considered high when using the *I*^2^ statistic (*I*^2^ = 98.88%). Egger’s regression test did not indicate the presence of publication bias (*p* = 0.41).

**Fig. 10:**
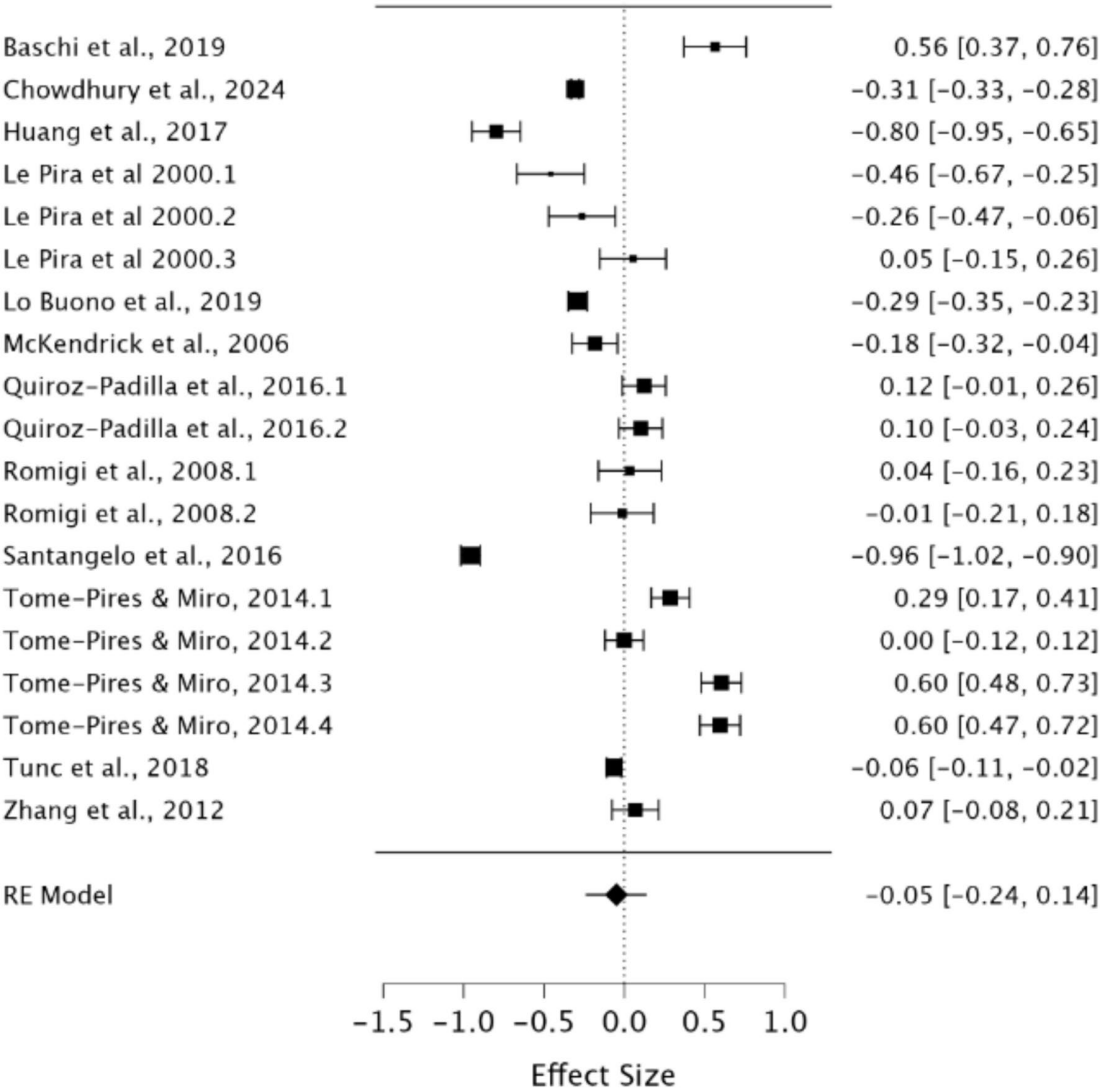
Forest plot of effect sizes and confidence intervals for verbal STM performance in migraineurs compared to healthy controls. *RE* Random effects, *Effect Size* Hedge’s g

**Fig. 11:**
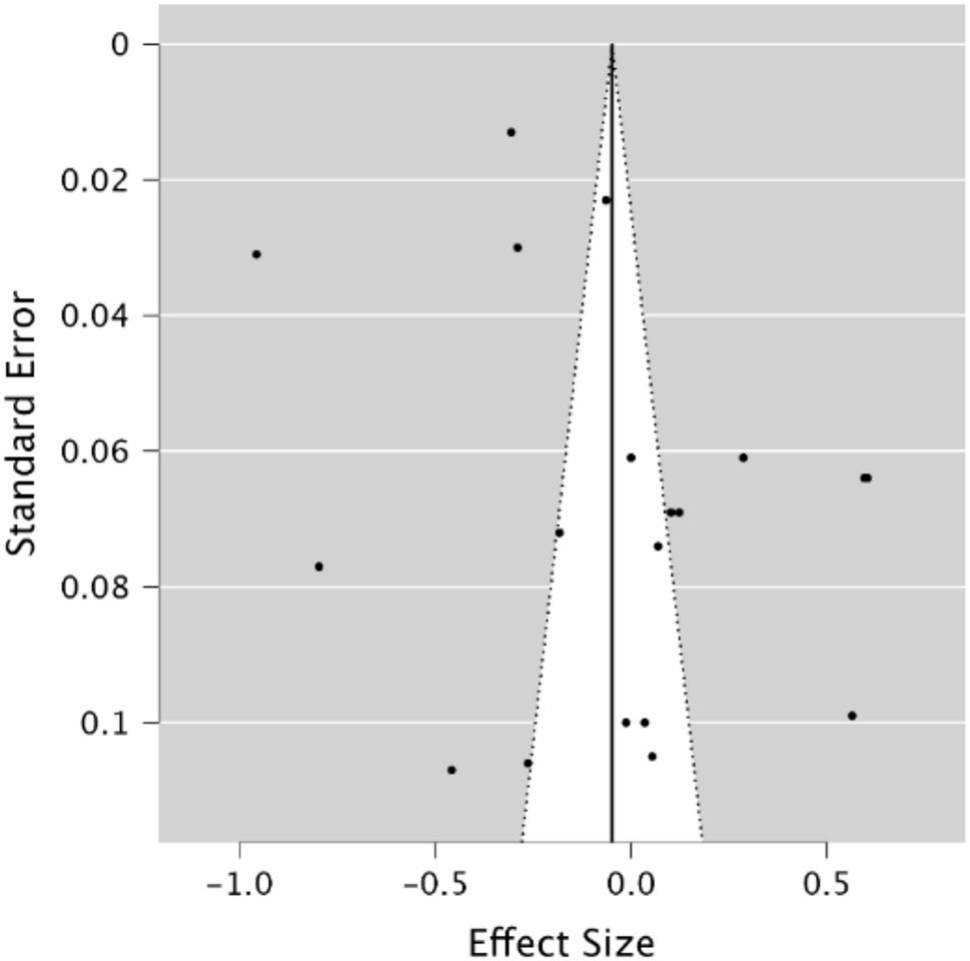
Funnel plot of potential publication bias for verbal STM performance in migraineurs compared to healthy controls

Finally, a random effects model was used to assess verbal STM in migraineurs compared to healthy controls. As observed in Fig. 10, no significant difference was present between groups demonstrating a small effect size (*g* = −0.05, 95% CI − 0.24–0.14, *p* = 0.62). The *Q* statistic was 1270.20 (*df* = 18) and was significant (*p* < 0.001), indicating significant heterogeneity. Heterogeneity was considered high when using the *I*^2^ statistic (*I*^2^ = 98.88%). Egger’s regression test and visual inspection of the funnel plot (Fig. 11) did not indicate the presence of publication bias (*p* = 0.37).

## Discussion

### Interpretation of the findings

This systematic review and meta-analysis examined STM and WM performance in adults with migraine. Across analyses, STM performance did not differ significantly between migraineurs and healthy controls, nor were differences observed by migraine subtype or memory modality. In contrast, a significant difference emerged for WM, indicating poorer WM performance in migraineurs relative to controls. Together, these findings suggest a dissociation between preserved short-term storage and compromised higher-order memory processes in migraine.

The absence of STM impairment indicates that the capacity to temporarily store information remains intact in migraineurs at a group level. STM tasks primarily assess passive maintenance of information over brief intervals and place relatively limited demands on attentional control or executive resources. Further interrogating these findings, analyses by migraine subtype did not reveal differential impairment in MwA or MwoA, aligning with previous work showing preserved cognition despite structural and functional brain differences relative to controls [37, 38]. These patterns are compatible with the possibility that neuronal plasticity and network reorganisation may help maintain cognitive functioning in migraine. Moreover, no differences emerged when STM was examined by modality. This suggests that visual and verbal STM are similarly preserved in migraine, even though aura manifestations can include visual, speech, and sensory disturbances [8]. The present findings therefore offer no evidence that aura-related phenomena translate into lasting modality-specific foundational memory deficits.

In contrast, the observed WM impairment points to difficulties in the manipulation, updating, and control of information held in the conscious store [12]. This performance pattern aligns with models that note WM as a capacity-limited system that is sensitive to fatigue, pain-related cognitive load, and fluctuating neural efficiency [14, 18, 20]. This finding also suggests that brain networks supporting executive control (e.g. frontally mediated systems) may be more vulnerable than those supporting basic memory storage [25]. Importantly, the finding that WM, but not STM, is affected provides a more nuanced account of migraine-related cognitive dysfunction.

This distinction is reflected in the cognitive demands of standardised memory tasks. STM measures such as digit span forward primarily assess storage capacity under minimal interference, whereas WM tasks, including digit span backward and the paced auditory serial addition test [39], place higher demands on attentional control, processing speed, and mental effort. The presence of a significant WM effect in the current meta-analysis suggests that migraine-related cognitive difficulties may be most evident under conditions requiring active manipulation and sustained cognitive engagement. This distinction may help explain why individuals with migraine often report difficulties in real-world cognitive functioning despite preserved performance on simpler memory tasks.

This dissociation between intact STM and impaired WM has important implications for understanding the commonly reported experience of brain fog in migraine. Brain fog is frequently described as difficulty concentrating, mental slowness, and reduced ability to manage complex or multi-step tasks [5]. These experiences are commonly reported in everyday contexts that place sustained cognitive demands on individuals with migraine and are not easily captured by brief or low-demand neuropsychological assessments. From this perspective, brain fog may be experienced as difficulty sustaining cognitive performance under increased mental effort rather than as an absolute failure of memory or immediate attention. This framing helps reconcile preserved STM performance on standardised tests with the substantial subjective cognitive complaints reported by individuals with migraine, and is consistent with evidence from other clinical populations in which cognitive complaints are more closely tied to effortful demands than to simple memory retention [40].

Importantly, the absence of objective short-term memory impairment does not negate the lived experience of brain fog. Previous work has demonstrated that subjective cognitive complaints can be dissociated from performance on neuropsychological tests [41]. The present findings suggest that whilst basic short-term storage capacity is preserved in migraineurs, difficulties may emerge at the level of working memory, particularly for tasks requiring active manipulation, sustained attention, or cognitive control. Brain fog may therefore reflect episodic or phase-specific disturbances in cognitive efficiency rather than a global or enduring memory deficit. Many STM tasks place relatively low demands on executive resources [13], which may explain preserved performance between attacks. In contrast, standardised memory tests may still fail to fully capture the fluctuating, real-time cognitive challenges reported by individuals with migraine in everyday contexts.

Taken together, these findings indicate that brain fog in migraine is unlikely to reflect a breakdown of core short-term memory capacity. Instead, brain fog may be better conceptualised as a state-dependent disruption in cognitive efficiency, particularly affecting higher-order memory processes that rely on attentional control and mental effort. This framework integrates subjective cognitive complaints with objective evidence of selective WM vulnerability and provides a more nuanced account of migraine-related cognitive dysfunction. Finally, it is important to note that WM impairment is unlikely to fully account for brain fog, which is a multifactorial phenomenon likely involving interactions between attention, fatigue, pain, and affective factors.

### Limitations

This study has several limitations that should be considered. Heterogeneity was high across analyses, reflecting variability in migraine characteristics, participant demographics, and cognitive assessment methods. Although subgroup analyses were conducted, residual heterogeneity remained, which is common in meta-analyses of complex clinical populations [42]. Additionally, whilst STM and WM tasks were classified according to dominant conventions in the literature, some measures involve overlapping cognitive processes, which may contribute to variability in effect sizes. Nonetheless, the consistent pattern of preserved STM alongside impaired WM supports the robustness of the observed dissociation.

Certain confounding factors exist as further limitations. Studies predominantly assessed migraineurs during the interictal phase. Likely an ethical consideration given the severity of symptoms that are present during the ictal phase. However, this means the relationship between the active symptoms of migraine and cognitive performance cannot be wholly confirmed. Similarly, most studies do not require migraineurs to cease medication or stipulate medication use as exclusion criteria for the same ethical consideration. This highlights another potential confounding factor, as it is understood that medication can impact cognitive performance [43]. Finally, the majority of participants involved in the included studies were female. While this reflects the known prevalence patterns of migraine [2], rather than recruitment bias, it precluded examination of potential sex-specific effects on memory performance as most studies did not present sex-specific data. Sex differences have been observed both in migraine presentation and in aspects of cognitive functioning [29, 44], and future research with sufficient representation of males or sex-stratified analyses is needed to clarify whether short-term or working memory outcomes differ by sex in migraine populations.

### Implications and future directions

The present findings have important implications for clinical practice and future research. The preservation of STM may provide reassurance that basic memory storage is not compromised in migraine outside of attacks. However, the presence of WM impairment suggests that higher-order cognitive processes supporting everyday functioning may be vulnerable. Clinicians and educators may, therefore, consider strategies that reduce cognitive load, such as breaking tasks into smaller steps, allowing additional processing time, and minimising multitasking demands. From a research perspective, future studies should prioritise WM and executive-function measures and examine their relationship with subjective reports of brain fog across different phases of the migraine cycle.

Future research should extend the evidence base on WM and other cognitive domains, including executive function, and processing speed, to determine whether these more complex functions domains also map onto the experience of brain fog. Longitudinal and phase-specific designs that assess cognition across the migraine cycle, including ictal periods where feasible (e.g. via remote or brief digital assessments) [45], may clarify when and how cognitive difficulties arise. In addition, studies targeting specific migraine subtypes and symptom profiles, rather than collapsing across heterogeneous groups, may reveal subgroups who are more vulnerable to cognitive disruption. Finally, incorporating standardised measures of subjective cognitive complaints alongside objective tests would help link perceived brain fog to quantifiable outcomes and inform person-centred management strategies.

## Conclusion

This systematic review and meta-analysis indicates that short-term memory performance is preserved in adults with migraine, whereas working memory shows a selective impairment relative to healthy controls. These findings suggest that migraine is not associated with a global memory deficit, but rather with vulnerability in higher-order memory processes that require active manipulation and sustained cognitive control. At the same time, the reality of subjective brain fog should not be dismissed. By distinguishing between preserved foundational memory storage and impaired working memory, the present review helps reconcile objective cognitive findings with lived experience. Integrating objective and subjective perspectives will be essential for refining our understanding of migraine-related cognitive symptoms and for developing interventions that support everyday functioning and quality of life for people with migraine.

## Supplementary Information

Below is the link to the electronic supplementary material.

Supplementary file1 (DOCX 22 KB)

## Data Availability

The datasets generated and/or analysed during the current study are available in the Open Access at La Trobe (OPAL) repository, 10.26181/30929591.

## Abbreviations

MwoA: Migraine without aura
MwA: Migraine with aura
STM: Short-term memory
WM: Working memory
JASP: Jeffrey’s amazing statistics program
PRISMA: Preferred reporting items for systematic reviews and meta-analyses
